# Case studies of the winds in the urban area of Hong Kong – Microclimate station observations and high resolution numerical simulations

**DOI:** 10.1016/j.heliyon.2024.e37865

**Published:** 2024-09-12

**Authors:** K.W. Lo, P.W. Chan, K.K. Lai, S.P.W. Lau, Z.H. Zhao

**Affiliations:** a Hong Kong Observatory, Hong Kong, China; b Shenzhen Polytechnic University, Shenzhen, China

**Keywords:** Microclimate station, Smart lamppost, Urban meteorology, Computational fluid dynamics

## Abstract

Hong Kong, renowned for its densely packed urban areas, poses unique challenges for understanding the effects of buildings on local meteorological conditions. To address this, the Hong Kong Observatory has started building a network of urban meteorological monitoring stations since 2017 for monitoring, analysing and studying urban microclimate. This paper presents an observational and numerical study focusing on wind measurements obtained from wind sensors installed on two smart lampposts in Tsim Sha Tsui, a major urban area in Hong Kong. Two representative high wind conditions in Hong Kong, Super Typhoon Saola in 2023 and a strong monsoon case characterized by prevailing easterly winds, are considered. With the use of high resolution computational fluid dynamic simulations, major features of actual observations can be reproduced. This suggests that district scale or even street scale weather services could be possible in the future with sufficient computational power.

## 1 Introduction

With rapid urbanization occurring globally, many cities are increasingly defined by high-rise buildings and dense construction. The complex urban morphology often results in complicated flow patterns, hindering the understanding and prediction of local meteorological conditions. The associated wind hazards, including wind shear, turbulence, and gusts, can significantly impact public safety, particularly during extreme weather events such as tropical cyclones and intense monsoons. To better understand the influence of the complex urban morphology, a dense network of in situ measurements is necessary to capture the intricacies of urban airflow. For a recent review about microclimate measurement in tropical cities, readers can refer to Ref. [1]. Hong Kong, being a densely populated city characterized by its unique topography and high-rise buildings, serves as an ideal city for conducting such a study.

For monitoring the winds over Hong Kong and timely warning of high winds, the Hong Kong Observatory (HKO) has operated a network of over 30 anemometers within the territory. These anemometers are mainly set up at strategic locations with relatively open terrain, such as outlying islands, mountain tops, airport, etc. Nevertheless, the increasing demand for urban meteorology necessitates a shift toward more localized observations and forecasts that reflect the conditions experienced by residents [2].For this purpose, HKO has started building a network of urban meteorological monitoring stations. As a start, a network of HKO microclimate stations is gradually set up within the territory. A review of the site selection and case studies of these microclimate stations could be found in Ref. [3]. In addition, HKO also participated in a pilot scheme project by The Government of the Hong Kong Special Administrative Region (HKSARG), in which HKO selected suitable smart lampposts for installing meteorological devices to extend the urban meteorological observations. Monitoring the winds in urban areas during the passage of tropical cyclone (TC) is essential to assess the potential damage impact brought by the TC. Apart from TC, intense surge of northeast monsoon could also bring gale force winds to Hong Kong [4]. Thus, the present paper would mainly focus on wind measurements from the wind sensors of smart lampposts under two rather representative high wind conditions in Hong Kong, including one tropical cyclone case (Super Typhoon Saola in 2023) and a strong monsoon case with strong easterly winds prevailing over Hong Kong.

Apart from observations, forecasting of the winds over the urban area is also a subject of active pursuit in HKO. For this purpose, a mesoscale meteorology urban model is nested with a computational fluid dynamics (CFD) model for studying winds around buildings. Such studies are initiated at first because of building-induced windshear and turbulence at the Hong Kong International Airport ([5]). In the current study, such model coupling is extended to simulate the winds over a major urban area in Hong Kong, namely, Tsim Sha Tsui (TST), the downtown of Kowloon, Hong Kong, because of the installation of a dense network of microclimate stations and smart lamppost meteorological sensors over there.

Several recent studies have demonstrated the efficacy of using CFD for assessing wind conditions in urban areas during TC events. For instance, a Weather Research and Forecasting (WRF)/Large Eddy Simulation (LES) hybrid model was utilized to examine the impact of densely built environments in Osaka City on urban wind gusts during Typhoon Jebi [6]. The study revealed that maximum surface-level wind gusts could reach 60–70 m/s, comparable to wind speeds of the height of about 300 m. Sensitivity experiments were conducted with both realistic and idealized building arrangements in Kyoto City in Ref. [7], the result indicated that variability in building heights and complex urban layouts significantly enhanced surface wind gustiness. In Ref. [8], the urban wind environment in Kowloon, Hong Kong during Typhoon Haima was studied using CFD simulations with a k-omega turbulence model. The study incorporated local-scale observations into the CFD domain through tendency assimilation. Similarly, urban winds environment in Kolwoon during Typhoon Kammuri, with a focus on the K11 Building, was studied using nested LES model in Ref. [9]. In Ref. [10], an embedded LES model was used to simulate the winds at Tongji campus in Shanghai, China during Typhoon Hagupit, highlighting a reduction in computational costs compared to full LES simulations.

The present study employs a similar approach by using mesoscale NWP simulation to provide realistic boundary conditions for the CFD simulations. Our focus is on comparing the CFD simulations with measurement data from a dense network of microclimate stations in TST. The result indicates that the current setup could effectively capture the high spatial variability of urban winds in densely built environments. Furthermore, a sensitivity experiment is conducted to explicitly investigate the impact of high-rise building on pedestrian level winds, enhancing our understanding of wind dynamics in urban settings.

## 2 Method

### 2.1 Urban meteorological monitoring stations

In recent years, there has been an increasing interest in the world meteorological community in urban climate study. In addition, members of the public call for higher spatial resolution weather forecast, not only down to the district level but may be even to the street level. In this connection, the HKO started setting up a network of urban meteorological monitoring stations in urban areas of Hong Kong since 2017 for monitoring, analysing and studying urban microclimate.

HKO adopted market-available all-in-one full-type configuration compact weather stations, as well as collaborated with a start-up company to develop new microclimate measurement instruments utilizing the micro-electromechanical systems (MEMS). The market-available all-in-one full-type weather stations are able to provide quality air temperature, relative humidity, wind speed and direction data through third-party calibration certificate.

In addition to HKO's own microclimate stations, HKO also participated in the Multi-functional Smart Lampposts Pilot Scheme coordinated by the Office of the Government Chief Information Officer (OGCIO) of the HKSARG. Through the project, HKO selected representative locations for installing market-available all-in-one meteorological devices at suitable smart lampposts in urban areas.

As of March 2024, HKO has established 30 microclimate stations on its own, and with OGCIO's assistance, established over 60 sets of urban meteorological monitoring stations on smart lampposts in urban areas. Meteorological sensors of microclimate stations and smart lampposts altogether provided near real-time observation in street levels of Hong Kong.

The present paper will include the wind observation from two sets of meteorological devices in smart lampposts in TST, namely DF0563 in Nathan Road and DF1400 in TST East (S1). Each set consists of two identical meteorological devices at the height of 3 m and 10 m from the ground respectively. The meteorological devices adopted are Gill MaxiMet GMX500 compact weather stations with individual calibration certificate traceable to United Kingdom Accreditation Service (UKAS). Wind speed and direction are measured by ultrasonic wind speed and direction sensor. Data of 1-min mean wind speed and 10-min mean wind speed in every minute have been utilized in the current study.

### 2.2 Numerical simulation

**Fig. 1:**
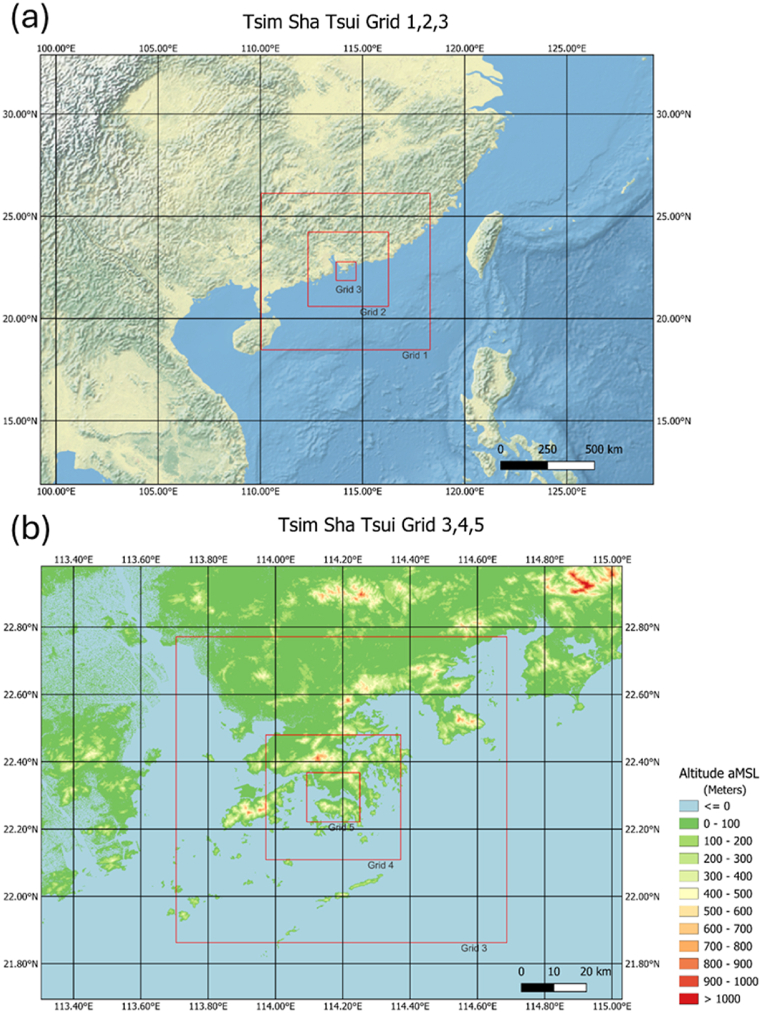
The 1st, 2nd and 3rd nested domain of RAMS simulation. (a) and the 3rd, 4th and 5th nested domain of RAMS simulation (b).

**Fig. 2:**
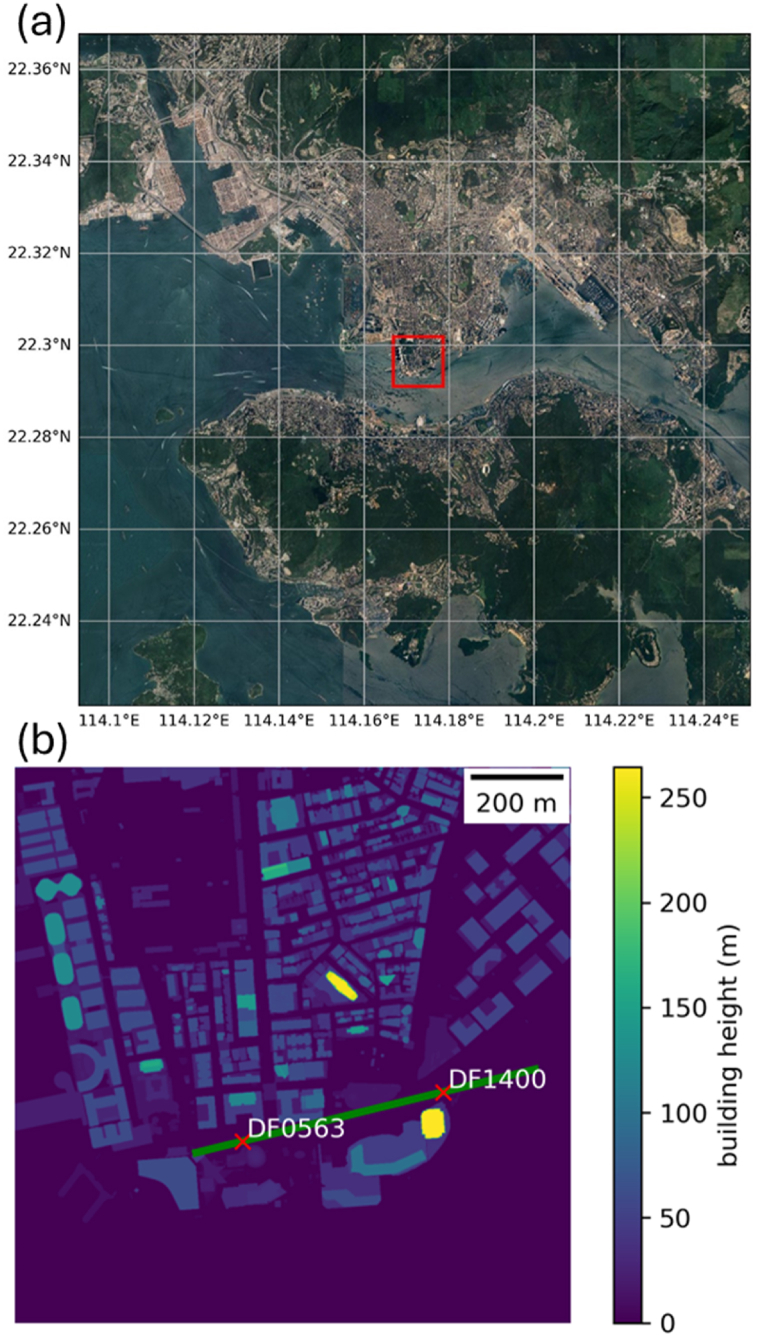
The domain of PALM simulation denoted by red rectangle. The boundary represents the 5th nested domain of RAMS simulation (a). Representation of building heights in PALM (b).

In this study, urban winds in the TST area are simulated by CFD method. The initial and boundary conditions of CFD model are provided through one way coupling from a mesoscale numerical weather prediction model, The Regional Atmospheric Modelling System (RAMS). The simulation set up of RAMS follows the approach outlined in Ref. [11] with Deardorff turbulence parameterization scheme [12] and large eddy simulation mode used. RAMS is configurated with five nested domains, with resolutions of 25 km, 5 km, 1 km, 200 m, and 40 m, respectively. The model domains of RAMS are shown in Fig. 1 (Fig. 1(a) for the first three domain and Fig. 1 (b) for the last three domain). The innermost domain is centred over Tsim Sha Tsui (TST), the area of interest. The first vertical grid has a size of 75m. A stretch ratio of 1.08 is applied and the maximum vertical grid spacing is limited to 2000m. The innermost domain of RAMS is then coupled with the CFD model, PALM in this study. The 5th nested domain of RAMS simulation with the domain of PALM simulated denoted by red rectangle is shown in Fig. 2(a).

PALM is a parallelized LES model which solves non hydrostatic filtered, incompressible Navier-Stokes equations in Boussineseq-approximated form. LES explicitly resolves the larger energy-containing eddies while the smaller subgrid-scale (SGS) turbulence is modelled via parameterization. In PALM, 1.5-order turbulent closure scheme based on the Deardorff model is used for the parameterization [12]. The closure scheme assumes that the SGS energy transport is proportional to the local gradients of the resolved quantities. The proportional constant, SGS eddy coefficient of momentum, is determined from the SGS turbulent kinetic energy and SGS mixing length. For more details about PALM and its governing equations, please refer to Refs. [[13], [14], [15]]. Fifth-order upwind momentum advection scheme [16] and third-order Runge-Kutta time stepping scheme [17] are used. Adaptive time stepping scheme is used such that the time step is automatically determined based on the Courant Friedrichs Levy criterion. The horizontal resolution in PALM simulation is 2m and the vertical resolution is initially 2m, with vertical stretch ratio of 1.08 applied starting from 50m. The maximum vertical grid size is limited to 12m. Synthetic turbulence generation is also applied [18,19]. The simulation domain of PALM, along with the building heights within the domain, is illustrated in Fig. 2(b). The locations of the two microclimate stations used in this study, DF1400 and DF0563, are also indicated.

PALM and RAMS are coupled through one-way off line nesting where initial condition and time varying boundary conditions for PALM are generated from RAMS. The detail of coupling between RAMS and PALM is discussed in Ref. [5]. Table 1 shows a summary of the computational setting of RAMS and PALM. The CFD simulation had been repeated for coarser horizontal resolutions of 3m and 4m for one of the test cases. The results from these coarser simulations generally agreed with the 2m simulation, with the 3m simulation being more aligned with the 2m simulation (S3).

**Table 1:** Summary of computational settings for RAMS and PALM simulation.

	RAMS (domain 1, 2, 3)	RAMS (domain 4, 5)	PALM
Horizontal resolution	25 km, 5 km, 1 km	200m, 40m	2m
Number of horizontal grids	37x37, 82x82, 102x102,	207x207, 407x407	600x600
Time step	10s, 5s, 2.5s	5/6s, 5/18s	adaptive
Initial vertical resolution	75m	75m	2m
Initial and boundary conditions	Derived from NCEP GFS/nested from outer domain	Nested from outer domain	Derived from RAMS
Turbulence model	Smagorinsky [32]	Deardorff [12]	Deardorff [12]

The simulated wind speed and direction at the smart lampposts DF0563 in Nathan Road and DF1400 in TST East could be obtained from PALM by simple interpolation due to its fine vertical resolution. However, as the initial vertical grid size in RAMS is already larger than the heights of the measurement devices in the smart lampposts, a log wind profile is applied to estimate the corresponding wind speed in RAMS data by assuming a roughness length of 2m [20].

## 3 Results and discussion

### 3.1 Case study: Super Typhoon Saola

Super Typhoon Saola brought about significant impact to Hong Kong during late August and early September of 2023. An observation study and a forecasting aspect study of Saola could be found in Refs. [21,22] respectively. The isobaric chart in the evening of September 1, 2023, is presented in S2(a). Saola was very close to Hong Kong at that time and wind force up to hurricane was recorded in the territory.

**Fig. 3:**
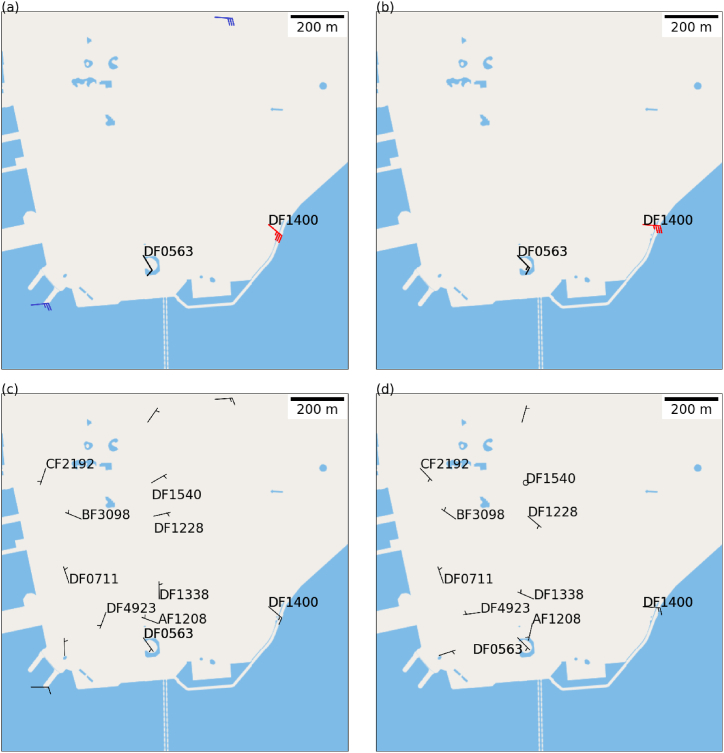
Observations from conventional anemometers and microclimate stations at 3m (a) and microclimate stations at 10m (b) at TST area in Hong Kong at 11:00pm, September 1, 2023, local time. Observations from conventional anemometers and microclimate stations at 3m (c) and microclimate stations at 10m (d) at 12:00 nm, March 10, 2024, local time. By March 10, 2024, more microclimate stations were installed in Tsim Sha Tsui.

The observations from microclimate stations at the height of 3 m and conventional anemometers at TST area in Hong Kong are given in Fig. 3(a) and the observations from microclimate stations at the height of 10 m are present in Fig. 3(b). Unfortunately, there were just a couple of microclimate stations at TST area at that time, namely, one at the Nathan Road (DF0563) and another one at the TST East area (DF1400). It could be seen that gale force winds were recorded at the site of more exposure to the east, namely, the TST East station, whilst the station along Nathan Road was surrounded by buildings and the winds were generally lighter over there. Kruskal-Wallis test indicates that the winds at these two microclimate stations follow different distributions, which suggests that the urban morphology have significant impact on the urban wind distributions.

For each station, the following wind-related parameters are simulated, namely, 10-min and 1-min mean wind speed, 10-min-mean wind direction. To quantify the fluctuations of wind speed, gustiness of the wind is considered. Similar to Refs. [23,24], gustiness, *G*, is calculated using the mean and standard deviation of 1-min mean wind speed over 30 min, *U*, as follow:

(1) $$\begin{array}{c} G=std\left(U\right)/mean\left(U\right)\text{.} \end{array}$$

At each station, the meteorological devices are two levels above ground, namely, 3 m and 10 m, are considered.

**Fig. 4:**
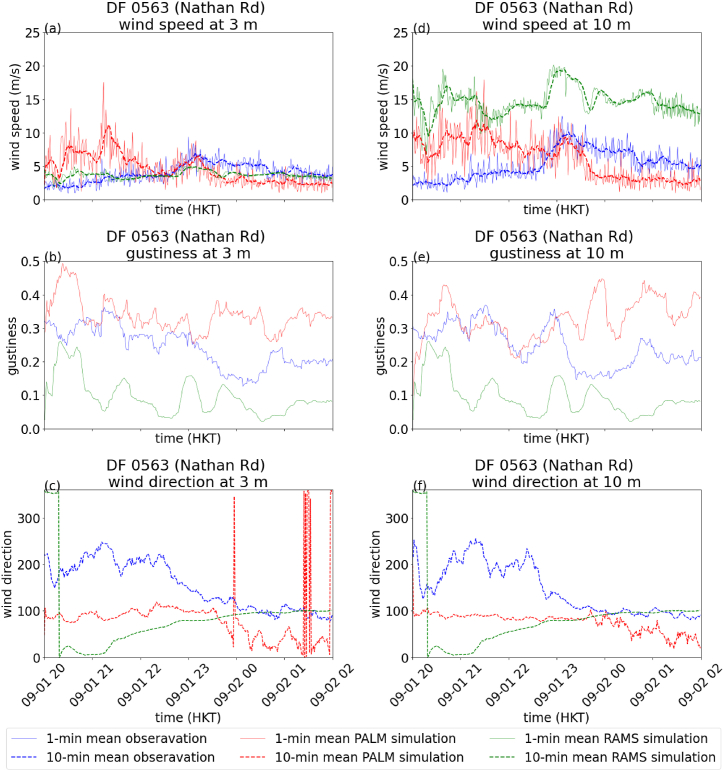
Comparison of measurement and simulation data from RAMS and PALM for DF0563 on September 1, 2023. 1-min (solid line) and 10-min (dashed line) mean wind speed at 3m (a), gustiness at 3m (b) and 10-min mean wind direction at 3m (c), 1-min and 10-min mean wind speed at 10m (d), gustiness at 10m (e) and 10-min mean wind direction at 10m (f).

The results of the Nathan Road station (DF0563) are shown in Fig. 4, with the root mean square error (RMSE) of 10-min mean wind speeds from the last 4 h of the simulation summarized in Table 2. In general, 10-min mean wind speeds from PALM simulation are consistent with the actual observations (Fig. 4(a) for 3m and (d) for 10m). On the other hand, larger error was observed for the RAMS simulation for measurement at 10m. The 10-min mean wind directions in the PALM simulation are consistent with those of the actual in the latter part of the simulations (Fig. 4(c) for 3m and (f) for 10m). The discrepancies in the earlier part of the simulation may be because of the representation of the structures of the buildings in TST area and the upstream area in the northerly wind conditions. For gustiness, the PALM simulation tends to over-estimate, which indicates that the representation of the buildings and roughness in the model require further refinement to achieve a more accurate simulation of the wind gustiness. On the other hand, gustiness is severely underestimated in the RAMS simulation even though large eddy simulation mode and Deardorff turbulence scheme is adopted (Fig. 4(b) for 3m and (e) for 10m).

**Table 2:** Root-mean-square-error (RMSE) and correlation for simulated 10-min mean wind speeds from RAMS and PALM compared with the measurement for the case of Super Typhoon Saola. Data from the last 4 h of the simulation was used for the calculation.

Microclimate station	DF1400		DF0563	
Device height	3m	10m	3m	10m
RMSE (RAMS)	11.29	2.38	1.13	8.83
RMSE (PALM)	5.30	4.88	1.79	3.14

**Fig. 5:**
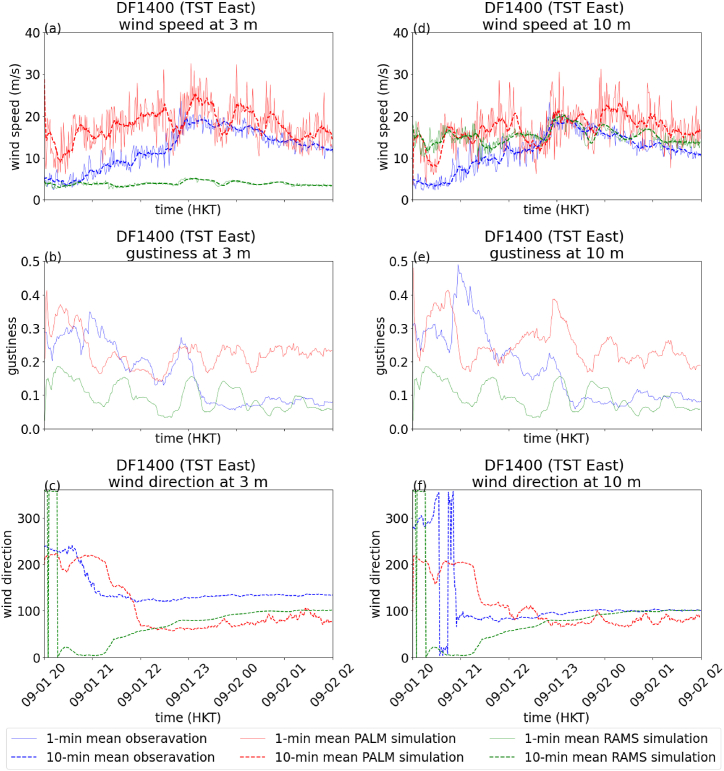
Comparison of measurement and simulation data from RAMS and PALM for DF1400 on September 1, 2023. 1-min (solid line) and 10-min mean (dashed line) wind speed at 3m (a), gustiness at 3m (b) and 10-min mean wind direction at 3m (c), 1-min and 10-min mean wind speed at 10m (d), gustiness at 10m (e) and 10-min mean wind direction at 10m (f).

The results of the TST east station (DF1400) are shown in Fig. 5. The RMSE of 10-min mean wind speed is also summarized in Table 2. The conclusions about the PALM simulation of 10-min mean wind speed (Fig. 5 (a) for 3m and (d) for 10m) and gustiness (Fig. 5(b) for 3m and (e) for 10m) are similar to those of the Nathad Road station. However, the RAMS simulation shows significant error for the measurement at 3m, which is related to the vertical structure of the near surface boundary layer winds and will be further discussed below (Fig. 5(a)). Furthermore, the simulation results of PALM for 10-min mean wind directions are more consistent with actual observations at this microclimate station, which is likely due to reduced influence of man-made structures on the wind direction as this station is closer to the coastline (Fig. 5 (c) for 3m and (f) for 10m).

By comparing the observation at 3m and 10m, it can be seen that the wind speeds at 10m are higher than that at 3m for DF 0563 (Fig. 4(a) and (d)), while the wind speeds are comparable, and sometimes even larger at 3m than 10m for DF 1400 (Fig. 5(a) and (d)). Such behaviour is also observed in PALM simulation. However, it could not be reproduced from RAMS data as the assumption of log wind profile restricted the wind speed to be monotonically increase with height. The application of log wind profile is doomed to fail to reproduce the wind structure of near surface boundary layer at DF1400 no matter what roughness length was chosen. The higher wind speed near surface around DF 1400 might be attributed to the downwashing effect of a tall building, Victoria Dockside, at its vicinity (Fig. 2(b)). For DF0563, the building heights around it are more uniform despite it is surrounded by more man-made structures.

**Fig. 6:**
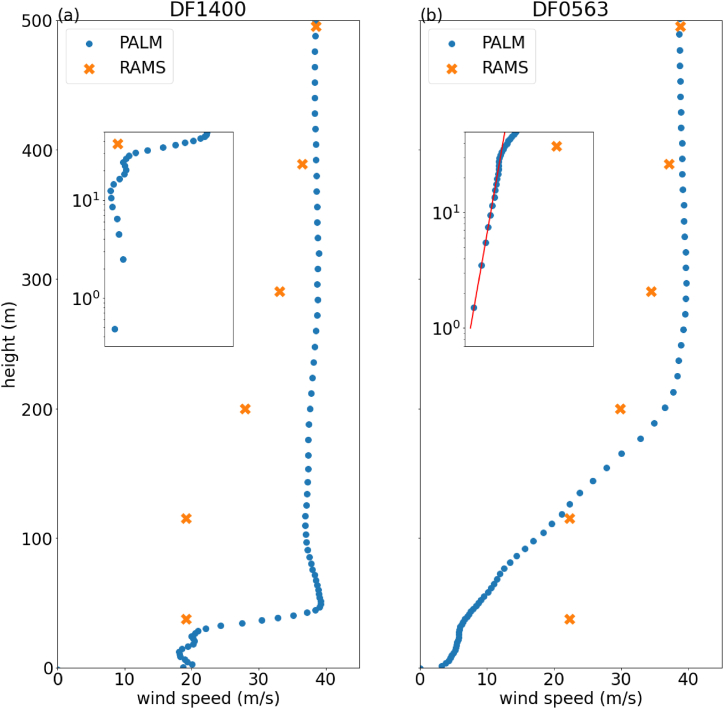
Plot of vertical profile of temporal averaged horizontal wind speed at two microclimate station (a) DF1400 and (b) DF0563 on 1st Sept 2023. The zoomed-in plots showed the vertical profiles at the bottom 50m with log scale in both x and y axes.

Fig. 6 shows the vertical profiles of temporally averaged horizontal wind speeds for the two microclimate stations, DF1400 (Fig. 6(a)) and DF0563 (Fig. 6(b)), derived from RAMS and PALM simulations. The data from the last 4 h of the simulation was used for calculating the time average. Despite RAMS simulation having a horizontal resolution as fine as 40m (the distance between the two microclimate stations is about 450m), the vertical profiles at the two stations appear quite similar due to the lack of explicit building representation in RAMS simulation. For PALM simulation, while the vertical profiles of wind speed are similar above 300m, there are substantial differences underneath, especially near the surface. To better visualize these differences near the surface, zoomed-in plots for the bottom 50 m with both axes in log-scale are also provided in Fig. 6. At DF0563, the temporally averaged horizontal wind speed below 40 m can be well-represented by a power law function, with an exponent of 0.18 and an R-squared value of 0.995. At DF1400, the horizontal wind speeds exhibit a more complicated dependence on altitude. The wind speed reaches a local maximum at around 2.5 m above the surface, then decreases to a local minimum at approximately 15 m. It then undergoes another cycle of increase and decrease before a more rapid increase is observed between 30 m and 40 m, indicating a transition from the roughness sub-layer to inertial sub-layer. The complex wind profile at DF1400 with multi-layered structure of alternating increases and decreases in wind speed indicates the intricated influence of surrounding buildings. Such a detailed wind profile is difficult to be modelled by parametric or empirical formulas and a fine scale CFD simulation is necessary to fully capture it.

**Fig. 7:**
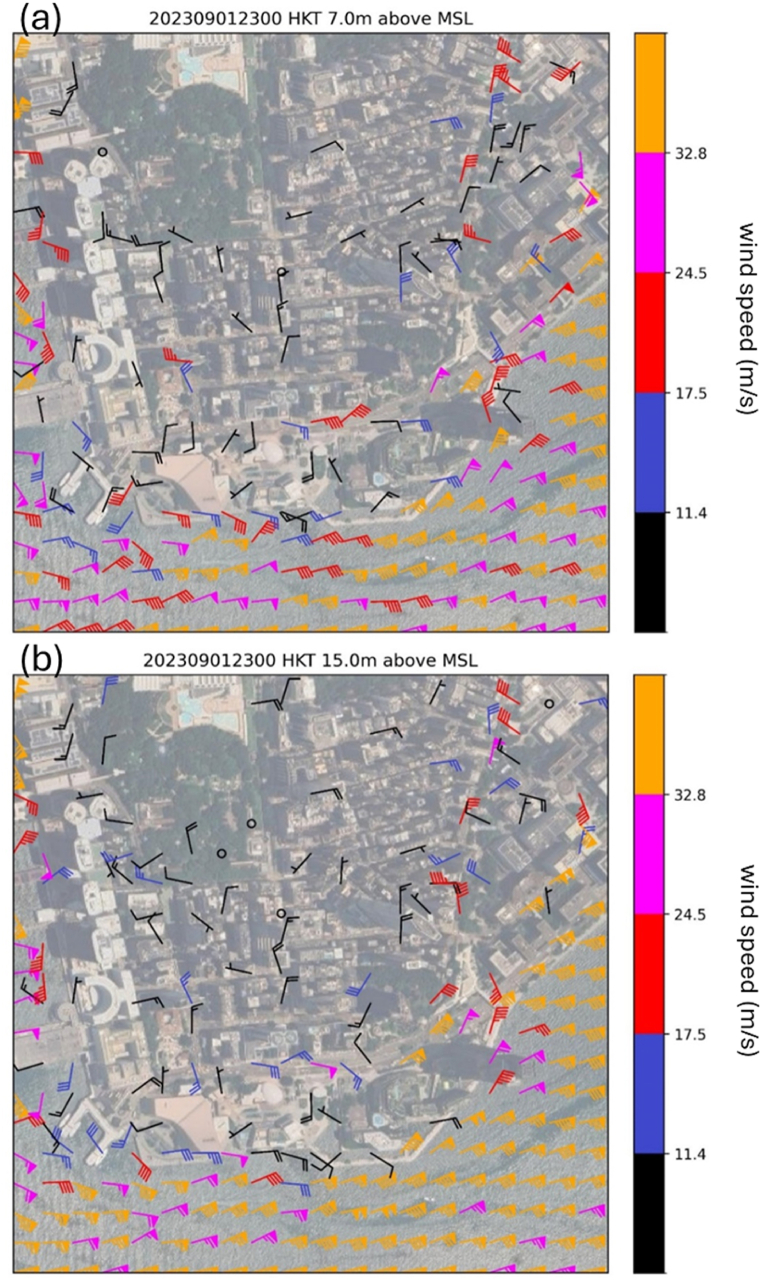
Simulated horizontal winds distribution over TST area at 11:00pm, September 1, 2023, at 7 m above mean sea level (a) and 15 m above mean sea level (b).

A snapshot of the wind distribution over TST area in the simulation is given in Fig. 7 for two heights above mean sea level, namely, those close to the 3 m (Fig. 7(a)) and 10 m (Fig. 7(b)) above local ground, for comparison with the actual observations in Fig. 3(a) and (b). Despite the limited number of microclimate and anemometer stations available, the simulated winds over the southern part of TST area resemble the observed winds pattern. On the eastern coast of TST, particularly along the streets, the simulation indicates that the 10-min mean wind speeds could reach hurricane force, as indicated by the orange color. Unfortunately, there were no real observations to validate the simulation results. If these hurricane force winds are accurate, it would have significant impact on the timely wind warnings to the pedestrians on the streets. Additionally, it is also observed that the western boundary of the simulation domain exhibited some unrealistic wind patterns, which is probably due to its close proximity to the buildings in the urban area. While a larger domain might solve this issue, this is constrained by the availability of the computational resources unfortunately. Nevertheless, the simulated winds over there are not important for the case study and weather warning operations.

### 3.2 Case study: strong monsoon

The surface isobaric chart of the case, namely, March 10, 2024, is shown in S2(b). A ridge of high pressure along the southeastern coast of China was bringing up strong easterly winds to Hong Kong. The surface observations at TST area are shown in Fig. 3(c) and (d). By March 10, 2024, more microclimate stations were installed in Tsim Sha Tsui. It is noteworthy that there were some light northerly winds along the Nathan Road (e.g. microclimate station DF1338) though the prevailing wind direction is easterly. The northerly wind to the north and the east to southeasterly winds to the south over the TST area may lead to local convergence, which is a favourable condition for the occurrence of localized urban heat island in the central part of TST in hot summer days (though it was not the case of the current study for March 10, 2024). This phenomenon is the primary motivation for this study, which is to investigate the origin of light northerly winds over some parts of Nathan Road when the prevailing winds is easterly.

**Fig. 8:**
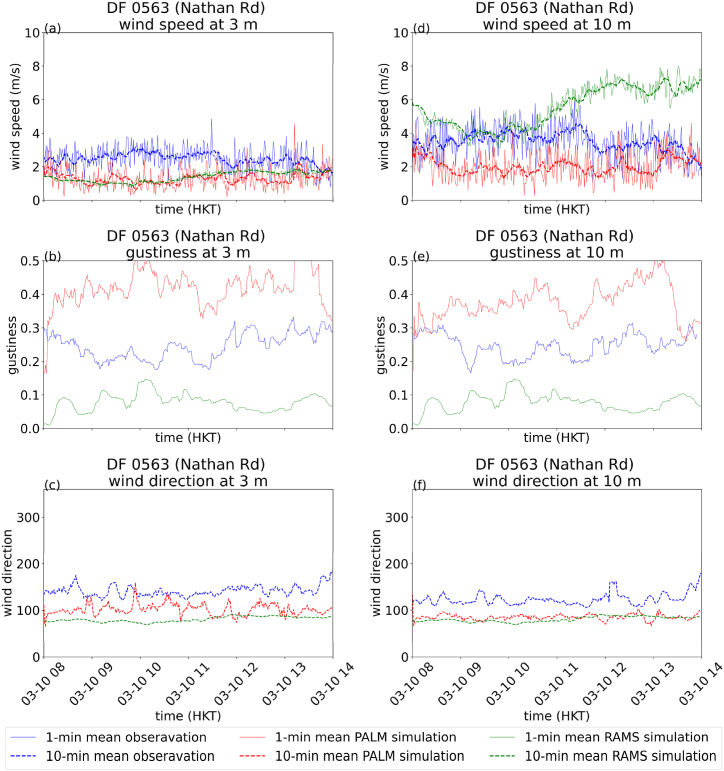
Comparison of measurement and simulation data from RAMS and PALM for DF0563 on March 10, 2024. 1-min (solid line) and 10-min (dashed line) mean wind speed at 3m (a), gustiness at 3m (b) and 10-min mean wind direction at 3m (c), 1-min and 10-min mean wind speed at 10m (d), gustiness at 10m (e) and 10-min mean wind direction at 10m (f).

**Fig. 9:**
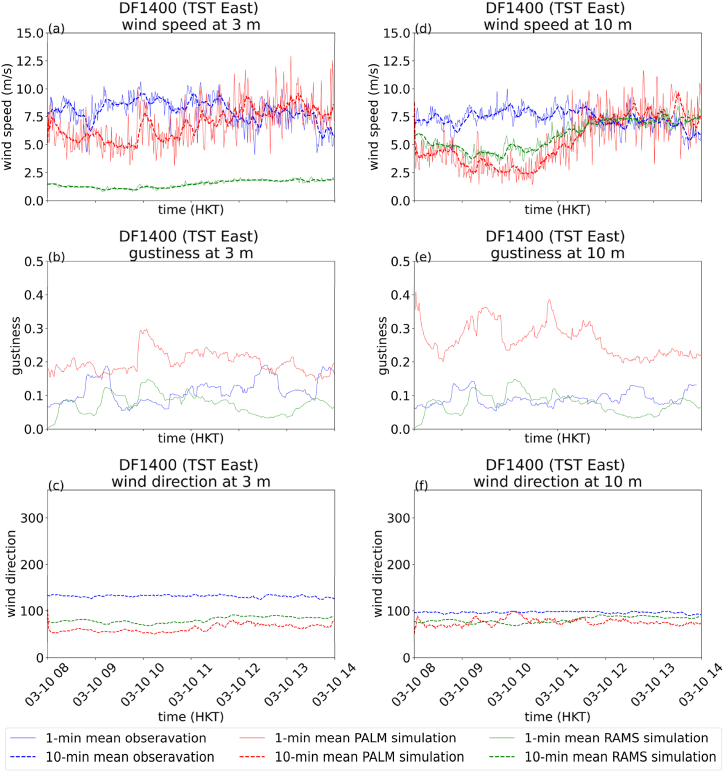
Comparison of measurement and simulation data from RAMS and PALM for DF1400 on March 10, 2024. 1-min (solid line) and 10-min (dashed line) mean wind speed at 3m (a), gustiness at 3m (b) and 10-min mean wind direction at 3m (c), 1-min and 10-min mean wind speed at 10m (d), gustiness at 10m (e) and 10-min mean wind direction at 10m (f).

Fig. 8, Fig. 9 show the time series of wind speed (Fig. 8(a) for DF0563 at 3m, 8(d) for DF0563 at 10m, 9(a) for DF1400 at 3m and 9(d) for DF1400 at 10m), gustiness (Fig. 8(b) for DF0563 at 3m, 8(e) for DF0563 at 10m, 9(b) for DF1400 at 3m and 9(e) for DF1400 at 10m) and wind direction (Fig. 8(c) for DF0563 at 3m, 8(f) for DF0563 at 10m, 9(c) for DF1400 at 3m and 9(f) for DF1400 at 10m) at the two microclimate stations DF0563 and DF1400. The RMSE for the 10-min mean wind speeds of RAMS and PALM are summarized in Table 3. It could be seen that the actual observations and PALM simulations at the two heights and the two locations are rather consistent with each other. In contrast, the RAMS simulation again shows significantly larger error for the wind speeds at 10m for DF0563 and 3m for DF1400 (Fig. 8, Fig. 9). The CFD simulations, which explicitly resolve the buildings, are adding values over pure mesoscale/microscale numerical weather predictions. In particular, the simulated wind speeds at a height of 3 m above ground appear to match rather well with the actual measurements. This suggests the potential development of urban scale/street level weather services, at least for the winds forecasting using CFD simulations. Similar to the case of Super Typhoon Saola, wind speeds are generally larger at 3m than 10m for DF 1400 in the measurement data, such a behaviour is also successfully reproduced by PALM simulation.

**Table 3:** Root-mean-square-error (RMSE) and correlation for simulated 10-min mean wind speeds from RAMS and PALM compared with the measurement for the case of Super Typhoon Saola. Data from the last 4 h of the simulation was used for the calculation.

Microclimate station	DF1400		DF0563	
Device height	3m	10m	3m	10m
RMSE (RAMS)	6.46	1.79	1.05	2.99
RMSE (PALM)	1.96	2.77	1.30	1.68

**Fig. 10:**
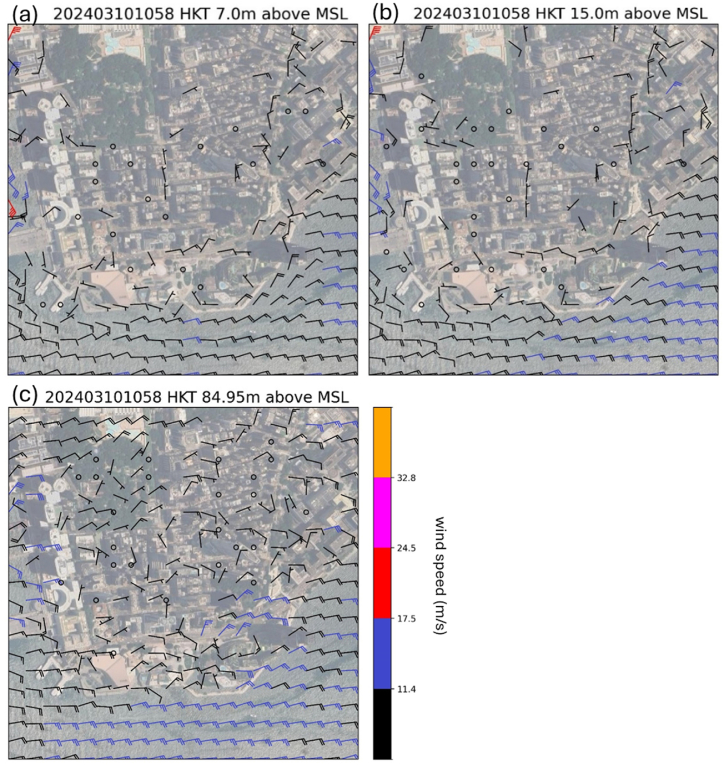
Simulated horizontal winds distribution over TST area at 10:58 a.m., March 10, 2024, at 7 m above mean sea level (a), 15 m above mean sea level (b) and 84.95 m above mean sea level (c).

Despite the prevail wind was easterly over Hong Kong, the wind directions over Tsim Sha Tsui exhibited significant spatial variation due to the complicated building arrangement. The distribution of simulated winds over TST area is shown in Fig. 10 (a) and (b) for 7m and 15m above mean sea level. Comparing with the actual observations in Fig. 3(c) and (d), though the winds are generally light in the TST area because of blocking and roughness of buildings, the wind directions are quite consistent with each other between the actual observations and numerical simulations. In particular, light northerly winds were successfully captured by the simulation over parts of Nathan Road. An examination of winds over the region at higher altitude is shown (Fig. 10(c)). The prevailing easterly winds appear to be distorted by the buildings, resulting in a board area of cyclonic flow (possibly similar to the lee low downstream of a mountain). The western side of this small cyclonic flow leads to light northerlies along the north-south oriented Nathan Road.

**Fig. 11:**
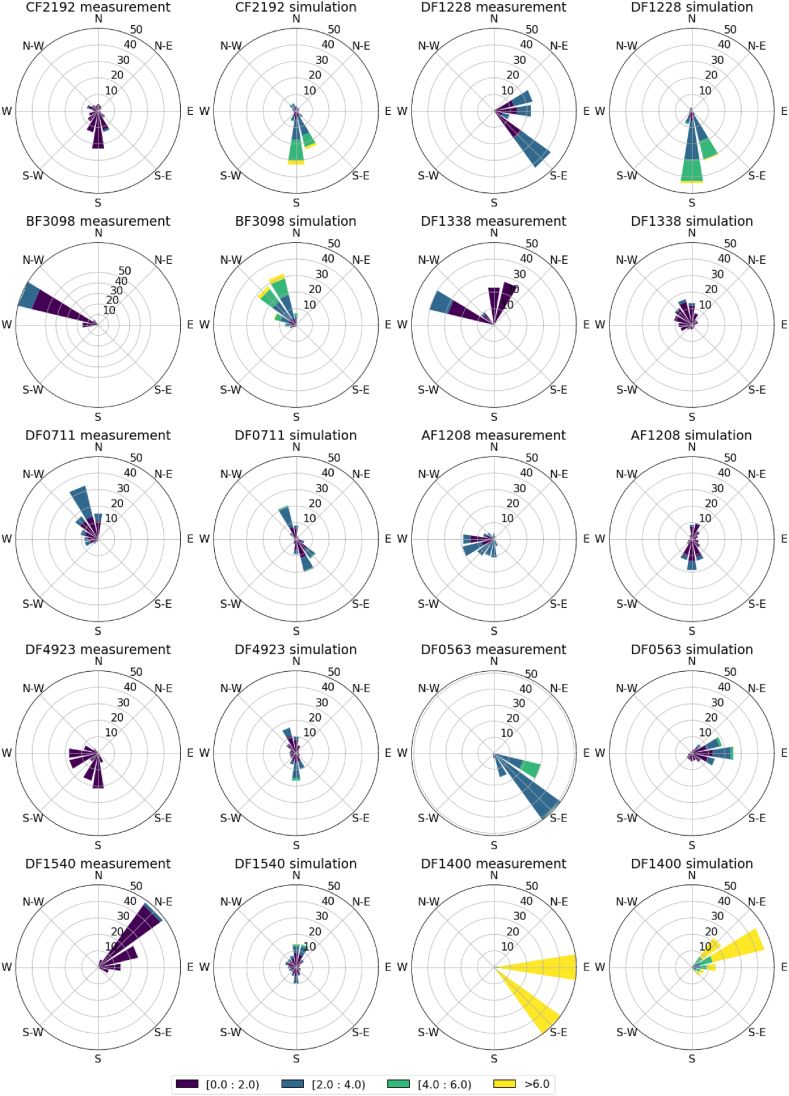
Wind rose for 10 microclimate stations for measurement and CFD simulation data on March 10, 2024.

Fig. 11 shows the plots of wind rose of DF1400, DF0563 and eight additional microclimate stations on March 10, 2024. For simplicity, winds at 3m and 10m are plotted on the same figure. The first and third columns show the measurement values while the second and fourth columns show the corresponding simulated values from PALM. The result demonstrates that the PALM simulation is generally able to capture the high spatial variation of urban winds within the small area around TST. For example, the southerly winds recorded at the CF2192 station along Canton Road, as well as the westerly component of the winds observed at station BF3098 along Kowloon Park Drive, are reproduced in PALM simulation. Furthermore, the CFD simulation successfully captures that the wind speeds are highest at DF1400 compared to the other microclimate stations. This shows the current simulation set up has reasonable skill in reproducing the urban winds in TST.

### 3.3 Effect of tall building on the urban winds

**Fig. 12:**
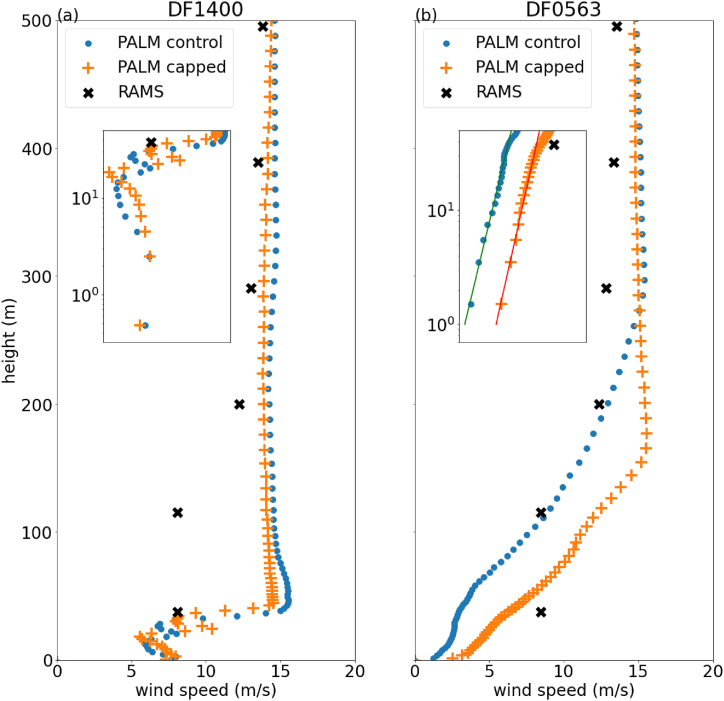
Plot of vertical profile of temporal averaged horizontal wind speed at two microclimate station (a) DF1400 and (b) DF0563 on March 10, 2024. The zoomed-in plots showed the vertical profiles at the bottom 50m with log scale in both x and y axes.

The vertical profiles of simulated temporal averaged horizontal wind at DF1400 and DF0563 for March 10, 2024 are plotted in Fig. 12(a) and (b) respectively. The black crosses represent the RAMS simulation, while the blue dots represent the PALM control simulation. The features of the vertical wind profile are roughly similar to the previous case of Super Typhoon Saola. At DF0563, a power law dependence is observed for the near surface winds, while at DF1400, a non-trivial vertical dependence is seen, with a transition from the roughness sublayer to the inertial sublayer occurring around 30–40 m.

To determine the potential impact of the nearby Victoria Dockside on pedestrian level winds, an additional CFD simulation was conducted with the height of Victoria Dockside capped at 50 m. The results are plotted in Fig. 12 using orange plus signs, labelled as "PALM capped". For DF0563, the power law dependence is again observed, but the wind speeds are roughly twice as large as the control simulation. At DF1400, the layered structure of wind speeds, with the transition from roughness to inertial sublayer, is reproduced. The pedestrian-level wind speed is slightly lower than the control simulation.

**Fig. 13:**
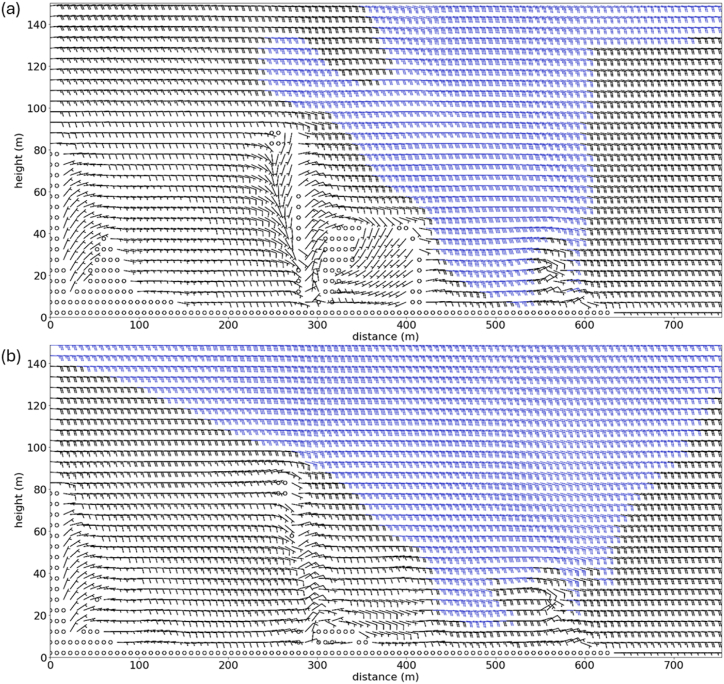
Vertical cross section of temporal averaged winds for control PALM simulation (a) and simulation where the height the Victoria Dockside is capped at 50 m (b). The plane of cross section is indicated by the green line shown in Fig. 2(b).

To better understand the difference in these two simulations, vertical cross section of winds for a plane passing through the two microclimate stations are plot in Fig. 13. The cross section is indicated by green line in Fig. 2(b), with smaller value on the x-axis representing location further to the west. For reference, DF0563 and DF1400 are located roughly at 100 m and 550 m along the x-axis respectively. In the control simulation (Fig. 13(a)), a recirculation region up to 50 m in height is observed on the leeward side of the Victoria Dockside building. However, when the height of Victoria Dockside is capped (Fig. 13(b)), this recirculation region is much smaller, extending only up to about 20 m above the surface. Due to the blockage caused by the larger recirculation region in the control simulation, the wind speed at DF0563, located further downstream, is much smaller compared to the "PALM capped" simulation. Furthermore, the comparison between Fig. 13a and b shows that the region of strong winds extends closer to the surface at region around Victoria Dockside when its height is not capped, suggesting that the stronger wind speeds at the pedestrian level in the control simulation is due to the downward transport of momentum from the high-rise building [6].

## 4 Discussion

The results of this study demonstrate that high-resolution CFD simulations can effectively reproduce key features observed by the microclimate stations, including the non-monotonic variation of wind speeds with altitude at DF1400 and the significant spatial variability across the TST area, which are absence in the mesoscale model due to the lack of explicit representation of buildings. To further quantify the accuracy of the simulation results, statistics comparison of the measured and CFD-simulated 10-min mean wind speeds for the two cases of Saola and strong monsoon are summarized in Table 4, Table 5. Data from the last 4 h of the simulation period are used in the analysis. For the case of Super Typhoon Saola, the CFD simulation shows good agreement with the measurement at DF0563 in term of the maximum of 10-min mean wind speeds, but the mean values exhibit slight underestimation. At DF1400, the simulated values tend to over-estimate the actual measurement. For the case of strong monsoon, the simulated values show better agreement with the measurement at DF1400. While the simulation exhibits a rather large percentage error at DF0563, this is due to the relatively small actual measurement values, which should have little impact on wind hazard assessment if the current setup was to be deployed operational in the future. The difference in error behavior at the two microclimate stations for these two cases suggests that the errors are not solely attributed to the representation of urban morphology or surface roughness alone, but also influenced by the imposed conditions from the mesoscale NWP model.

**Table 4:** Summary of statistics for 10-min mean wind speed of the measurement and CFD simulation for the case of Super Typhoon Saola. Data from the last 4 h of the simulation was used for the calculation.

Microclimate station	DF1400				DF0563			
Device height	3m		10m		3m		10m	
Wind speed (m/s)	mean	max	mean	max	mean	max	mean	max
Measurement	15.0	19.3	14.29	19.08	4.66	7.03	6.4	10.13
CFD simulation	19.17	24.71	18.04	22.52	3.61	6.84	5.12	10.54
Absolute error	4.17	5.41	3.75	3.44	−1.05	−0.19	−1.28	0.41
% error	28 %	28 %	26 %	18 %	−23 %	−3%	−20 %	4 %

**Table 5:** Summary of statistics for 10-min mean wind speed of the measurement and CFD simulation for the case of strong monsoon. Data from the last 4 h of the simulation was used for the calculation.

Microclimate station	DF1400				DF0563			
Device height	3m		10m		3m		10m	
Wind speed (m/s)	mean	max	mean	max	mean	max	mean	max
Measurement	8.0	9.59	7.42	9.24	2.45	3.09	3.47	4.57
CFD simulation	7.32	8.66	6.05	9.54	1.29	2.13	2.02	2.93
Absolute error	−0.68	−0.93	−1.37	0.3	−1.16	−0.96	−1.45	−1.64
% error	−9%	−10 %	−18 %	3 %	−47 %	−31 %	−42 %	−36 %

The simulations were performed using 1000 cores on a high-performance computing system. For the 6-h simulation periods, the total required time was about 58 h for the Super Typhoon Saola case and 22 h for the strong monsoon case. The difference in simulation times is due to the significantly higher wind speeds in the Super Typhoon Saola case, which requires a much smaller time step to satisfy the Courant-Friedrichs-Lewy condition. If such a NWP-CFD coupled model was to be deployed for real-time operation, the total simulation time would need to be reduced by an order of magnitude. This may be possible through technological advancements, such as the improvement in computing power of processors or the use of GPU to accelerate CFD simulations ([25,26]. Alternatively, the building-resolving microclimate simulations could be generated by the technique of super-resolution using convolutional neural networks similar to the proposed method in Refs. [27,28].In Ref. [28], it was reported that the total wall-clock time could be reduced to just 3.2 % of the original time. However, results from accurate fine-scale CFD simulation are still required to provide the necessary training data.

Apart from high-resolution simulations, another promising approach for forecasting wind speeds in urban areas is to utilize machine learning to train historical wind data for wind prediction at microclimate stations. For example, urban microclimate prediction was studied via artificial neural network, in particular multiple layer perceptron, using weather station data in Ref. [29] and long short-term memory network has been used in urban microclimate prediction in Ref. [30]. However, this approach would be limited by the availability and quality of historical data, which may not be available in rapidly changing urban environments. Furthermore, it is difficult for machine learning models trained with data at specific sites to predict locations where no measurement data exists, particularly in areas with unique or highly variable urban morphology. In contrast, high-resolution CFD simulations provide an estimation of wind conditions in regions lacking measurement data. Thus, high-resolution CFD simulations are still needed to fill in the gaps in data availability and could also be used as training data for urban microclimate prediction [31].

## 5 Conclusion

This study highlights the use of a network of microclimate stations and high resolution CFD simulations to study the complex wind patterns with urban environments, particularly TST in Hong Kong, through two representative high winds cases of tropical cyclone and intense monsoon. The simulations successfully reproduce the key features observed by the microclimate stations, including the non-monotonic variation of wind speeds with altitude and significant spatial variability due to complex urban morphology across the TST area. These findings suggest that the development of building scale weather services at street level could be possible when supported by a rather dense network of microclimate stations and sufficient computation power for high resolution CFD simulations. Moreover, a sensitivity experiment was conducted to assess the impact of a tall building on pedestrian-level winds. Interestingly, when the building height was capped, wind speeds in its immediate vicinity decreased, while wind speeds further downstream increased. This effect is attributed to reduced downward momentum transport and a smaller recirculation region, which led to less blockage of airflow to the downstream area when the building height is capped. The computational resources and time required for running the high-resolution CFD simulations and the feasibility for real-time forecasting, with the use of artificial intelligence, are discussed.

Additional case studies would be conducted to further validate the above points, especially tropical cyclone situations to observe and to simulate the high winds over the streets. More detailed statistical analysis between observations and simulations would also be performed when more case studies are completed.

## Data availability statement

Data that has been used in this manuscript is confidential.

## CRediT authorship contribution statement

**K.W. Lo:** Formal analysis. **P.W. Chan:** Writing – original draft, Conceptualization. **K.K. Lai:** Formal analysis. **S.P.W. Lau:** Data curation. **Z.H. Zhao:** Formal analysis.

## Declaration of competing interest

The authors declare that they have no known competing financial interests or personal relationships that could have appeared to influence the work reported in this paper.
