# Supplementary material for: Case studies of the winds in the urban area of Hong Kong – Microclimate station observations and high resolution numerical simulations
Source: Heliyon. 2024 Sep 12;10(18):e37865. doi: 10.1016/j.heliyon.2024.e37865 (PMC11417313; doi:10.1016/j.heliyon.2024.e37865)
Supplement: Multimedia component 1 [file mmc1.docx]

Supplementary Material


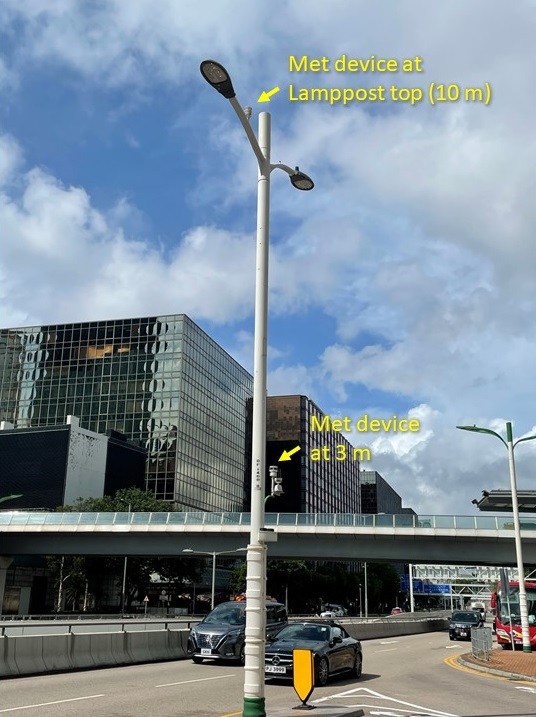


S1 The microclimate station DF1400 consisted of a set of two meteorological (Met) devices installed at the height of 3 m and 10 m from the ground on a Smart Lamppost in Salisbury Road, Tsim Sha Tsui, Kowloon, Hong Kong


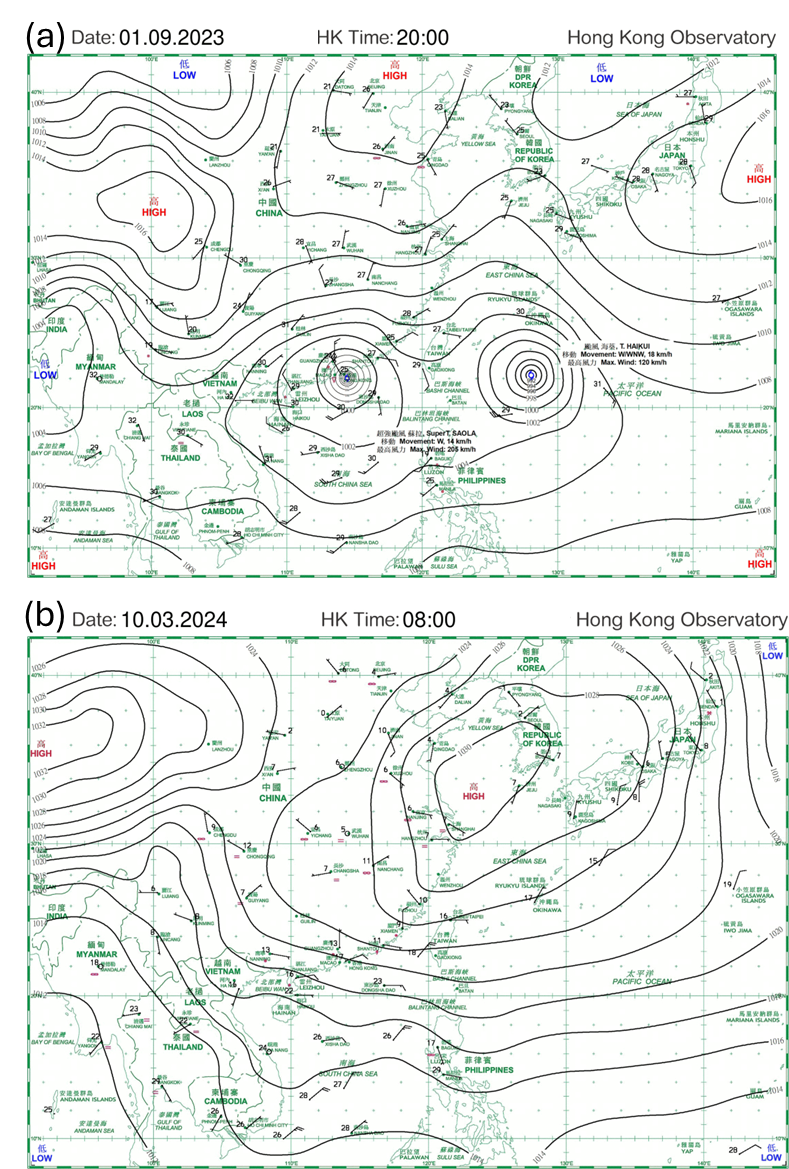


S2 The surface isobaric chart at 8p.m., 1 September 2023 local Time (+8 UTC) (a) and at 8a.m., 10 March 2024 local time (b).


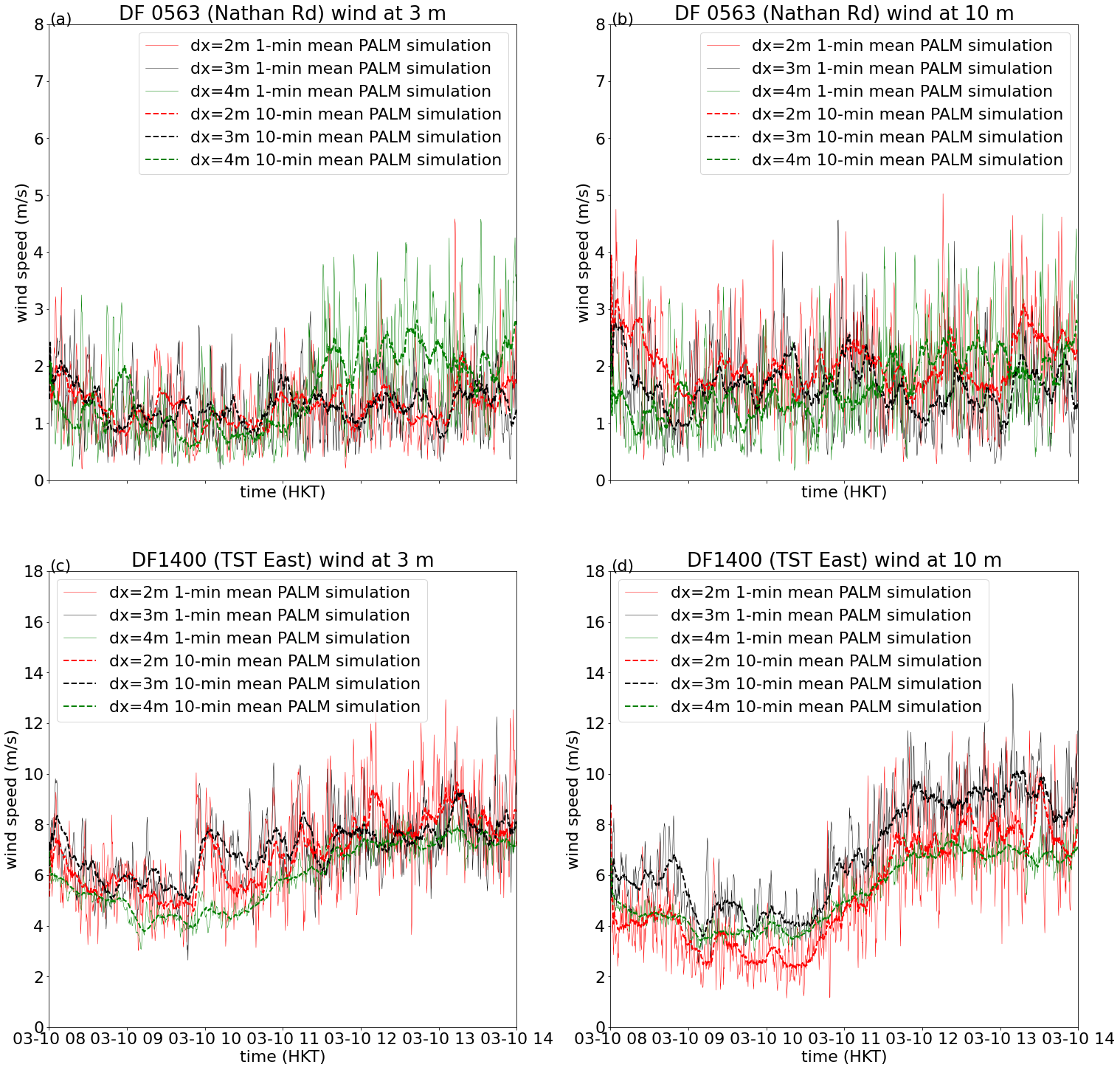


S3 Comparison of simulation result of horizontal wind speed on 10 March 2024 for different horizontal resolution for DF0563 at 3m (a); DF0563 at 10 m (b); DF1400 at 3m (c) and DF1400 at 10m (d).
